# The Western Dental Journal

**Published:** 1887-02

**Authors:** 


THE WESTERN DENTAL JOURNAL.—This new aspirant for favor has made its appearance in a very creditable style, appearance. The first number contains some valuable articles and if the succeeding numbers are made up of like material, this new journal will merit the patronage of the dental profession. Publishers: R. I. Pearson & Co., Kansas City, Mo. We trust that this increase in dental periodical literature indicates an increased interest in the members of the profession to advance with the science.

